# Therapeutic evaluation of video-assisted thoracoscopic surgery versus open thoracotomy for pediatric pulmonary hydatid disease

**DOI:** 10.1186/s13019-016-0525-9

**Published:** 2016-08-05

**Authors:** Jinshan Ma, Xiaolei Wang, Xaokat Mamatimin, Nuerlan Ahan, Kang Chen, Chuanliang Peng, Yongwei Yang

**Affiliations:** 1 People’s Hospital of Xinjiang Uygur Autonomous Region, Urumqi, Xinjiang 830001 People’s Republic of China; 2 Secondary Hospital of Shandong University, Jinan, Shandong 250000 People’s Republic of China

**Keywords:** Video-assisted thoracoscopic surgery, Thoracotomy, Pulmonary hydatid disease, Children

## Abstract

**Background:**

Hydatid disease is a severe and widespread human cestode infection, and in children, the lung is the most commonly infected organ. In current practice, the standard surgical procedure for the removal of pulmonary hydatid cysts is thoracotomy; therefore, we evaluated the efficacy and safety of video-assisted thoracoscopic surgery (VATS) to treat pediatric pulmonary hydatid disease. To our knowledge, this is the first and large sample comparative study of VATS and thoracotomy for pediatric pulmonary hydatid disease.

**Methods:**

In this study, we retrospectively reviewed 44 (61.1 %) pediatric patients who underwent VATS, and 28 (38.9 %) pediatric patients who underwent conventional thoracotomy from January 2005 to June 2012. Perioperative data, including basic characteristics of patients, the length of hospital stay, intraoperative blood loss, thoracic intubation indwelling time, and complications were compared between VATS and thoracotomy in 72 children with pulmonary hydatid disease.

**Results:**

VATS was found to be a safe technique for the treatment of pediatric pulmonary hydatid disease, with zero intraoperative deaths. In the VATS and thoracotomy groups, the hospital stay durations were 10.50 ± 1.20 days and 17.30 ± 2.75 days, respectively, and occurrence rates of complications were 9.1 % (4/44) and 17.9 % (5/28), respectively. The hospital stays were shorter and the hospitalization costs was reduced for the patients who underwent VATS compared with conventional thoracotomy (*P =* 0.001). Although no statistically significant difference in the recurrence rates (*P =* 0.958) and complication incidence (*P =* 0.273) between the two surgical groups was observed, less intraoperative bleeding, shorter thoracic intubation indwelling time and reduced postoperative pain were observed in the patients who underwent VATS (*P =* 0.001).

**Conclusion:**

Our study demonstrates the feasibility and safety of VATS for pediatric pulmonary hydatid disease treatment, providing a practice-changing concept for the treatment of this disease in the community. VATS can be a promising therapeutic tool, by overcoming many of the drawbacks of thoracotomy, and can be used as an alternative to thoracotomy for selected pediatric patients.

## Background

Hydatid disease, which is caused by the tapeworms *Echinococcus granulosus* and *Echinococcus multilocularis*, is also known as echinococcosis or hydatidosis [1]. Echinococcosis is endemic to South America, Alaska, Canada, New Zealand, Australia, India, and the Middle East, with an estimated incidence between 1/50,000 and 1/20,000 individuals [2]. In Turkey, hydatid disease is endemic to the southeastern parts of the country, with an incidence of approximately 2 to 12 of every 100,000 individuals [3, 4]. However, that the disease is widespread in northwestern China due to the geographical environment, lifestyle, and cultural background. In China, the Xinjiang Uygur Autonomous Region has a high incidence rate of this disease (8.7–28.4/100,000) compared to other areas in China [3, 5–7], and the disease has become a serious public health problem in this region.

Hao and colleagues [5] have described the etiology, occurrence and pathogenic mechanisms related to echinococcosis in great detail. Although parasites can affect various organs of the body, echinococcosis generally targets the liver and lungs. In adults, the lung (18–35 %) is the second most common location for hydatid disease after the liver (50–70 %) [8]. However, in children, the lung is the primary infection site, with hydatid cysts occurring in the lungs and liver in 64 % and 28 % of pediatric cases, respectively [9, 10]. The primary modes of transmission in children include contact with dogs, drinking contaminated water and the ingestion of contaminated foods. Therefore, the diagnosis and treatment of pulmonary hydatid disease are very important in pediatric patients.

With the development of thoracoscopic techniques over the past decades, minimally invasive surgery has become a feasible approach for the treatment of pulmonary hydatid disease. Compared to thoracotomy, thoracoscopic surgery has some limitations. However, video-assisted thoracoscopic surgery (VATS) only involves a minor incision, which reduces the incidence of postoperative infection and complications, allowing for a fast recovery period. There has been a trend towards the gradual adoption of thoracoscopic surgery for the treatment of pulmonary hydatid disease [6, 11–16]. However, to our knowledge, there are few studies about the detailed advantage of VATS compared with thoracotomy in pediatric pulmonary hydatid disease. In this study, we, for the first time, systematically compared various parameters between thoracotomy and VATS such as the length of hospital stay, intraoperative blood loss, thoracic intubation indwelling time, and complications. The aim of this study was to evaluate the advantage of VATS in the treatment of pediatric pulmonary hydatid disease, and share our experience of treating pediatric pulmonary hydatid disease with VATS.

## Methods

### Patients

Children were included in the VATS group if they met the following inclusion criteria: the lesion was located in the lung periphery; no more than two lesions; no obvious adhesion of the lesion to the pleural cavity; and the patient and family agreed to accept VATS. The patient was excluded if any of the following conditions were met: the lesion was located in the hilum pulmonis and extended to the main or segmental bronchus; the lesion showed obvious adhesion to the pleural cavity or lutation; three or more unilateral lesions; or the patient or family did not agree to accept VATS.

A total of 76 children presenting with pulmonary hydatid disease were enrolled in our study from January 2005 to June 2012. Of these children, 44 underwent VATS, and 28 underwent conventional thoracotomy. Furthermore, 2 of the enrolled patients chose not to undergo surgery, and 2 patients were misdiagnosed with pleural effusion. The baseline characteristics of the patients enrolled in this study are shown in Table 1. These cases included 47 males and 29 females, with an average age of 13 years (range, 4 to 18 years). Prior consent from all patients and approval from the local Institutional Research Ethics Committee were obtained.

**Table 1:** Baseline data of the VATS and thoracotomy groups

Parameter	VATS (*n =* 44)	Thoracotomy (*n =* 28)	*P* value
Age			
Mean ± SD	9.80 ± 5.50	11.16 ± 6.34	
Range	4 ~ 15	5 ~ 18	0.397
Ethnicity			
Han	9	8	
Minority	35	20	0.429
Lesion site			
Unilateral	40	25	
Bilateral	4	3	0.821
Casoni test			
Positive	36	23	
Negative	5	4	0.755
Body mass index (kg/m2)	25.35 ± 3.15	25.65 ± 4.30	0.367
Diameter of lesion (cm)	3.20 ± 0.13	3.25 ± 0.35	0.196
Operation time (min)	99.90 ± 10.70	114.45 ± 15.20	0.143
Blood loss levels (ml)	8.80 ± 1.40	18.30 ± 3.46	0.001^a^
Hospital stay (d)	10.50 ± 1.20	17.30 ± 2.75	0.001^a^
Course of disease (months)	2.72 ± 1.20	2.19 ± 2.17	0.095
Pain scores	1.12 ± 0.52	1.69 ± 0.60	0.001^a^
Intubation indwelling time(d)	2.11 ± 0.16	5.32 ± 1.01	0.001^a^
complication incidence (%)	9.1(4/44)	17.9(5/28)	0.273
Recurrence (n)	3	2	0.958
Hospitalization costs (RMB/Thousand)	8.22 ± 1.66	11.52 ± 3.74	0.001^a^

^a^statistically significant

### Symptoms and auxiliary examinations

**Fig. 1:**
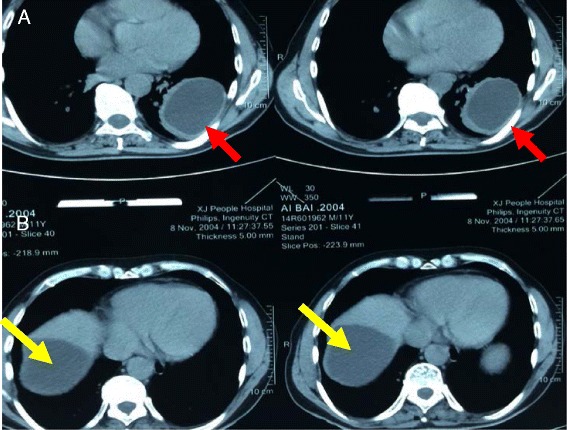
Computed tomography scan showing spherical opacity indicating the presence of a hydatid cyst in the left lower lung lobe (**a**, *red arrow*) and right liver (**b**, *yellow arrow*) of the same patient

In this study, 64 children (84.2 %) were from pastoral areas and had a long history of close contact with domestic animals or sheep farming. Common presenting symptoms included shortness of breath, cough, chest pain, intermittent watery and purulent sputum, hemoptysis and fever (38–40 °C) (Table 2). Oval or spherical opacities were observed in the lungs by chest computed tomography (CT) scanning. The cysts were located in the right lung in 34 cases, in the left in 35 cases and bilaterally in 7 cases. Upper abdominal CT scans and abdominal ultrasonography were performed to detect liver cysts, and liver cystic lesions were found in 23 cases (Fig. 1). The subcutaneous Casoni test had a positive rate of 86.7 % (59/68), and the indirect hemagglutination and convection immunity tests had positive rates of 83.9 % (47/56) and 65.5 % (36/55), respectively.

**Table 2:** Major clinical manifestations in symptomatic patients

Clinical finding	No. patients (%)
Cough	41(53.9 %)
Shortness of breath	12(15.8 %)
Sputum	31(40.8 %)
Chest pain	22(28.9 %)
Hemoptysis	9(11.8 %)
Fever	5(6.6 %)

### Surgical approach

Seventy-two pediatric patients aged 4 to 18 years with pulmonary hydatid cysts underwent surgery at the Department of Thoracic Surgery of the People’s Hospital of Uygur Autonomous Region by Jinshan Ma and his team. Patients were placed in the lateral decubitus position for most of the operation. Thirty minutes before the operation, the patient was administered 0.01 mg/kg atropine, 3–5 mg of dexamethasone, 3–5 μg/kg fentanyl, 2 mg/kg propofol, and 0.5–1.0 mg/kg atracurium for anesthesia induction. Anesthesia was maintained intraoperatively with inspiration of 1–2 % isoflurane and 5–10 μg/kg/min atracurium; supplemental fentanyl (5 μg/kg) was administered if anesthesia depth became insufficient. Intraoperative blood gas monitoring was performed. Surgery was performed under general anesthesia, with double-lumen endotracheal intubation (patient age ≥ 12 years) or deep single-lumen contralateral endobronchial intubation (patient age ≤ 11 years), unilateral lobar ventilation, or manual control respiration with monitoring.

We administered one-lung ventilation anesthesia via a single lumen endotracheal catheter interposed with the following precautionary measures. First, stable anesthesia induction and dosage, careful and precise handling of the trachea cannula, minimal airway pressure, and the induction of bronchus secretion to prevent the breaking of the hydatid were performed during the surgery. Second, the endotracheal catheter size (in mm) was selected based on the following formula: inner diameter (ID) = [age (years)/4] + 4. Endotracheal catheter location was determined via stethoscopy. Third, the catheter location was modulated if hypoxemia occurred, and if no improvements in bronchus secretion, low tidal volume and high frequency ventilation were observed, the one-lung ventilation method was abandoned, and the catheter was moved to the main trachea to enact the two-lung ventilation model to ameliorate the oxygen concentration. Fourth, extra-flow hydatid fluid may cause anaphylactic shock and death; therefore, the accurate detection of the blood pressure (BP), electrocardiogram (ECG), saturation of peripheral oxygen (SpO2), partial pressure of end-tidal carbon dioxide (PETCO_2_) values was essential. However, after the development of an allergy, anti-anaphylactic treatment must be quickly administered (e.g., iv epinephrine).

### VATS

**Fig. 2:**
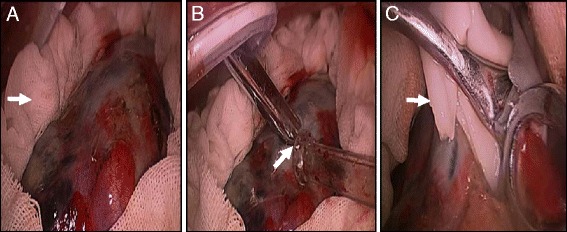
**a** Netting soaked with hypertonic saline solution was used to create a dike shape surrounding the lesion (*white arrow*). **b** Absorber determined the grey area for the puncture point (*white arrow*). A port for manipulation with an incision protector was located in the top left corner (*blue arrow*). **c** The hydatid cyst was dislodged by the oval window clamp after handling (*white arrow*)

After a standard prep and drape, a 1-cm incision was made in the 6th or 7th intercostal space in the midaxillary line to create an access port for observation. Then, a 2- to 3-cm incision was made in the 4th intercostal space in the anterior axillary line, or according to the location of the cystic lesion, to create an operative port. Another utility incision was made in the 7th or 8th intercostal space in the posterior axillary line. After single-lung ventilation and lung collapse, the three incisions were protected with an incision protector (Fig. 2b, blue arrow). Tissue surrounding the hydatid cyst was covered with gauze that had been moistened with 10 % hypertonic saline. The gauze formed a dike shape to prevent inadvertent implantation of scolices or daughter vesicles (Fig. 2a). Two closed-circuit aspirators were prepared and used to aspirate the hydatid cavity and around the lesion, respectively (Fig. 2b, white arrow). The cyst was removed with ring forceps after needle aspiration (Fig. 2c). Bronchial fistulas were carefully stitched up with absorbable sutures. Before the cyst was removed, the cyst cavity was managed with 10 % hypertonic saline for 5 min after aspiration.

The choice of surgical technique depended on the location, size, and intact state of the hydatid cyst (Table 3). For relatively large superficial cysts, a technique of needle aspiration plus cystotomy plus partial pericystectomy and residual cavity drainage was used. For cysts located deep inside the lung parenchyma, the technique was needle aspiration plus cystotomy plus capitonnage. For relatively small superficial cysts, enucleation was performed. Residual cavity and lung damage were treated with conservative methods such as pericystectomy, which is always associated with bronchial fistula suturing, capitonnage of the cavity or by adapted lung resections such as wedge resection, segmentectomy, and lobectomy. The surgical intervention was completed following chest tube placement into the residual cavity and the costophrenic localization of the pleural space (double catheter indwelling).

**Table 3:** Operative techniques employed in 72 patients

Operative technique	VATS group, N			Thoracotomy group, N		
	NA	Cys	En	NA	Cys	En
Additional palliative procedures						
Pericystectomy(partially)	14	6	3	8	2	2
Capitonnage	4	5	3	3	1	1
Additional radical procedures						
Wedge resection	5	3	1	6	3	1
Segmentectomy	1	2	0	1	1	0
Lobectomy	1	0	0	1	0	0

Removal of the cyst by (NA) needle aspiration, (Cys) cystotomy, or (En) enucleation of the intact cyst

### Thoracotomic approach

A standard posterolateral thoracotomic incision was made at the 5th intercostal space and was 8–15 cm in length. The thoracic cavity was opened layer-by-layer, and the ribs were separated carefully with a thoracic retractor to avoid fracture. The incision was protected by towels moistened with hypertonic saline (10 %). Surgical techniques were the same as employed in the VATS group.

According to our previous experience with treating hydatid disease, albendazole treatment was applied in the two groups for the management of the children with multiple intrathoracic cysts or additional liver cysts. This treatment regimen involved 15–20 mg/kg albendazole daily for two 10-day courses with a 20-day rest period between courses. All cysts were subjected to histopathologic examination to confirm the diagnosis.

### Measured outcomes

After the operation, patients underwent a complete blood count test, blood sedimentation, chest x-ray, and abdominal ultrasonography. These tests were repeated 1 month, 3 months, 6 months, and 12 months after surgery. Follow up included a routine in-clinic consultation service or telephone call every 12 months thereafter. If imaging revealed a lesion on the affected side with features consistent with a hydatid (e.g., homogenous density), in the absence of evidence of other disease, then hydatid relapse was assumed to have occurred.

The intraoperative and postoperative observations, including hospital stay, intraoperative blood loss, thoracic intubation indwelling time, complication incidence, and relapse rate, were recorded in detail after the operation and were extensively compared between the two groups.

### Statistical analysis

All quantitative data are expressed as the mean ± SD, and Student’s t-test was used to compare the means of the different groups. Categorical data were described as absolute frequencies and analyzed by Pearson’s Chisquare test or Fisher’s exact test. SPSS 20.0 software (Chicago, IL, USA) was applied to analyze the data, and an alpha value of *P <* 0.05 was considered statistically significant.

## Results

No significant difference was observed regarding the cyst anatomical location between the children who underwent VATS (*n =* 44) and the children who underwent conventional thoracotomy (*n =* 28). Three to six weeks after surgery, a laparotomy was performed in the patients with hepatic cysts. The preferred surgical techniques included the enucleation of the cyst or cystotomy (Table 3) and the closing of the cyst cavity by capitonnage in several patients. Perioperative death was not observed in the two groups. No study subjects were excluded from the study due to the conversion in treatment approach to thoracotomy, and postoperative complications primarily included partial atelectasis.

The hospital stay time was 10.50 ± 1.20 d and 17.30 ± 2.75 d in the VATS versus thoracotomy groups, respectively. Furthermore, intraoperative blood loss levels were 8.80 ± 1.40 ml in the VATS patients and 18.30 ± 3.46 ml in the thoracotomy patients (*P =* 0.001). In addition, the thoracic intubation indwelling time was 2.11 ± 0.16 d versus 5.32 ± 1.01 d in the VATS and thoracotomy groups, respectively(*P =* 0.001). The complication incidence was9.1 % (4/44) in the VATS group and was 17.9 % (5/28) in the other group (*P =* 0.273). These results strongly show that VATS not only shortened the duration of hospital stays and reduced the level of bleeding during the operation but also shortened the thoracic intubation indwelling time. Relapse rates were also compared during the 3- to 10-year follow-up study. The follow-up rate in the VATS group was 88.6 % (39/44), and the rate was 89.2 % (25/28) in the thoracotomy group. Among the VATS patients, pulmonary hydatid recurrence occurred in three subjects at 20, 27 and 31 months following surgery, and this complication occurred in two subjects at 19 and 25 months after surgery in the thoracotomy group; no significant difference in the occurrence of this complication was found between the two groups.

## Discussion

Hydatid disease first became known with the discovery of *E. granulosus* by Batsch in 1786 and *E. multilocularis* by Leukart in 1863 [17, 18]. Pulmonary hydatid disease is caused by infestation of the lungs with echinococcus larvae. This zoonotic disease is endemic in many sheep- and cattle-raising areas in northwest China [6, 7, 13, 14], and it remains an important health hazard throughout the world [1, 2, 5]. In recent decades, enormous efforts have been made towards managing pediatric pulmonary hydatid disease by various means. Nevertheless, surgery remains the best curative option. Thoracoscopic treatment of the thoracic hydatid cyst was first introduced for pediatric patients in 1993 [19]. However, there have been only a few relevant publications about this subject in the literature to date.

In the literature, the mean time of VATS for pediatric Pulmonary Hydatid Disease was 75 min, and the mean hospital stay was 5 days [12]. A recent study conducted by Parelkar also showed that the average duration of VATS procedure was 150 min and average length of hospital stay was 4.5 days [15]. In our series, the mean time of VATS was 99.90 (99.90 ± 10.70) minutes and the mean hospital stay of VATS was 10.50 days (10.50 ± 1.20). Post-operative complications mainly as partial atelectasis and neumothorax influenced the duration of hospital stay, hospital conditions also have certain influence on the length of hospital stay. Furthermore, in our study, VATS was superior to conventional thoracotomy in terms of the hospital stay, intraoperative blood loss, thoracic intubation indwelling time, postoperative pain scores and hospitalization costs. In children, the minimized surgical trauma observed with VATS resulted in less invasion and a rapid recovery period. Thus, VATS group have less intraoperative blood loss, postoperative pain scores and thoracic intubation indwelling time, these factors also resulted in shorter hospital stay and less hospitalization costs. Our results were also supported by previously published articles that reported the incidence of atelectasis [6, 9], which was significantly reduced in the VATS group compared with the thoracotomy group. A limitation of our study included difficulty in following up with the patients due travel difficulties, preventing us from determining a precise and accurate relapse rate.

The World Health Organization published an excellent overview of treatment guidelines for echinococcal disease in 1996 [20], which stated that immediate surgery may be required in patients with impending cyst rupture, compromised vital organs, hemoptysis, infected cysts, infection due to obstruction and unmanageable pain [21]. In recent years, with the rapid development of VATS, traditional thoracotomy has been gradually replaced by VATS as the primary surgical technique employed to treat this disease. VATS, a minimally invasive surgical approach, is characterized by less trauma, a quick recovery period and few complications, resulting in the popularity of using this method to treat lung disease [6, 11, 12].

In our work, VATS is typically employed in combination with hybrid VATS (H-VATS) in which a small incision is made first and is then gradually expanded to complete VATS (C-VATS) [13]. For the successful treatment of pulmonary hydatid disease in children, the VATS techniques should include the following measures: [1] the implementation of strict guidelines involving the conversion to thoracotomy; [2] the preservation of lung tissue around the cyst during thoracic cavity planting; [3] the prevention of airway obstruction during the hypertonic saline injection; and [4] the suturing of the bronchial communication. Current treatment regimens include a complete resection of the cyst with maximum preservation of lung parenchyma, especially in children, whose residual lung parenchyma possesses a large expansion capacity. Conservative procedures include cyst removal after needle aspiration and partial pericystectomy or cystotomy, and enucleation of intact cysts. Occasionally, an enclosed residual cavity can cause partial atelectasis. Therefore, in cyst cavity management, the bronchial communication should be sutured with care and the grey-white pericystic layer should be respected. The residual cavity is opened to form a bowel shape and to prevent the occurrence of fusion and pneumatosis, which can result in serious complications such as empyema and bronchopleural fistula. The anatomical features of the lung and the negative pressure of the pleural cavity facilitate hydatid cyst growth into the peripheral lung and emergence of at least part of the cyst on the lung surface. During the operation, indwelling of a catheter in the residual cavity is often necessary. In this study, our cyst removal techniques often involved conservative procedures rather than radical procedures such as wedge resection, segmentectomy, lobectomy, and pneumonectomy. In addition, when the cyst is located in the periphery, wedge resection can efficiently and safely remove the cyst. Segmental resection is preferable if the cyst occupies the entirety of the involved segment, and lobectomies are chosen when large cysts cover greater than 60 % of the lobe. Pneumonectomy is rarely necessary and should only be used when the entire lung is affected and no salvageable pulmonary parenchyma remains.

## Conclusion

VATS is a safe and effective surgical approach in the treatment of pediatric pulmonary hydatid disease. VATS is minimally invasive, has a rapid post-operative recovery period, results in minimal scarring and causes minimal blood loss. Therefore, VATS can be a promising therapeutic tool, by overcoming many of the drawbacks of thoracotomy. VATS can be used as an alternative to thoracotomy, depending on the size and location of the lesion. As thoracoscopy requires single-lung anesthesia, a skilled anesthetist should be an integral part of the thoracoscopy team.

## Abbreviations

BP, blood pressure; CT, chest computed tomography; C-VATS, complete VATS; ECG, electrocardiogram; H-VATS, hybrid VATS; ID, inner diameter; PETCO2, partial pressure of end-tidal carbon dioxide; SpO2, saturation of peripheral oxygen; VATS, video-assisted thoracoscopic surgery

## References

[1] Isitmangil T, Sebit S, Tunc H, Gorur R, Erdik O, Kunter E (2002). Clinical experience of surgical therapy in 207 patients with thoracic hydatidosis over a 12-year. Swiss Med Wkly.

[2] Salih OK, Topcuoglu MS, Celik SK, Ulus T, Tokcan A (1998). Surgical treatment of hydatid cysts of the lung: analysis of 405 patients. Can J Surg.

[3] Dogan R, Yuksel M, Cetin G, Suzer K, Alp M, Kaya S (1989). Surgical treatment of hydatid cysts of the lung: report on 1055 patients. Thorax.

[4] Statistics H (1997). Republic of Turkey Ministry of Health.

[5] Hao W, Xu MQ. Practical study of hydatid disease. 1rd ed. Beijing: Sciencn Press; 2007.

[6] Alpay L, Lacin T, Ocakcioglu I, Evman S, Dogruyol T, Vayvada M (2015). Is Video-Assisted Thoracoscopic Surgery Adequate in Treatment of Pulmonary Hydatidosis?. Ann Thorac.

[7] Wu MB, Zhang LW, Zhu H, Qian ZX (2005). Surgical treatment for thoracic hydatidosis: review of 1230 cases. Chin Med J(Engl).

[8] Yalav E (1980). Surgical treatment methods of pulmonary cysts.

[9] Petrov DB, Terzinacheva PP, Djambazov VI, Plochev MP, Goranov EP, Minchev TR (2001). Surgical treatment of bilateral hydatid disease of the lung. Eur J Cardiothorac Surg.

[10] Cangir AK, Sahin E, Enon S, Kavukcu S, Akay H, Okten I (2001). Surgical treatment of pulmonary hydatid cysts in children. J Pediatr Surg.

[11] Findikcioglu A, Karadayi S, Kilic D, Hatiopoglu A (2012). Video-Assisted Thoracoscopic Surgery to Treat Hydatid Disease of the Thorax in Adults: Is It Feasible?. J Laparoendosc Adv Surg Tech A.

[12] Amine K, Samia B, Jamila C, Mohamed BB, Lassad S, Sana M (2014). Thoracoscopic treatment of pulmonary hydatid cyst in children: a report of 25 cases. Tunis Med.

[13] Guo R (2012). Jinshan Ma, Nuerlan. Treatment of 42 child Cases of pulmanary echinococcosis granulosus by excision of internal cyst though video-assisted thoracoscopic surgery. Chinese J Parasit Parasitic Dis.

[14] Yongwei Y (2011). Jinshan Ma, Nuerlan. Video-assisted thoracoscopic surgery for pulmonary echonococcus: Report of 53 cases. Chin J Min Inv Surg.

[15] Parelkar SV, Gupta RK, Shah H, Sanghvi B, Gupta A, Jadhav V (2009). Experience with video-assisted thoracoscopic removal of pulmonary hydatidcysts in children. J Pediatr Surg.

[16] Oak SN, Parelkar SV, Satishkumar KV, Pathak R, Ramesh BH, Sudhir S (2009). Review of video-assisted thoracoscopy in children. J Minim Access Surg.

[17] Moreno MJ, Casado N, Urrea-Paris MA, Rodríguez-Caabeiro F (2002). Could ivermectin have a synergic effect with albendazole in hydatidosis therapy?. Parasitol Res.

[18] Perez-Serrano J, Denegri G, Casado N, Rodríguez-Caabeiro F (1997). In vivo effect of oral albendazole and albendazole sulphoxide on development of secondary echinococcosis in mice. Int J Parasitol.

[19] Becmeur F, Chaouachi B, Dhaoui N, Kaabar N, Popperova N, Bientz J (1994). Video-assisted thoracic surgery of hydatid cysts at the lungs in children. J Chir.

[20] WHO Informal Working Group on echinococcosis (1996). Guidelines for treatment of cystic and alveolar echinococcosis in humans. Bull World Health Organ.

[21] Mawhorter S, Temeck B, Chang R, Pass H, Nash T (1997). Nonsurgical therapy for pulmonary hydatid cyst disease. Chest.

